# Molecular and Phylogenetic Characterization of *Onchocerca flexuosa* in Red Deer from South-Eastern Europe

**DOI:** 10.3390/pathogens15040344

**Published:** 2026-03-24

**Authors:** Ervin Martinuš, Ema Gagović, Adnan Hodžić, Daria Jurković Žilić, Relja Beck

**Affiliations:** 1 EAM Konzalting, Zagrebačka 20, 49253 Lobor, Croatia; zagorje.lov@gmail.com; 2 Department for Bacteriology and Parasitology, Croatian Veterinary Institute, 10000 Zagreb, Croatia; gagovic@veinst.hr (E.G.); jurkovic@veinst.hr (D.J.Ž.); 3 Centre for Microbiology and Environmental Systems Science (CMESS), Department of Microbiology and Ecosystem Science, University of Vienna, 1010 Vienna, Austria; adnan.hodzic@univie.ac.at

**Keywords:** *Onchocerca flexuosa*, red deer, nodular onchocercosis, molecular identification, South-Eastern Europe

## Abstract

*Onchocerca flexuosa* is a vector-borne filarial nematode infecting red deer (*Cervus elaphus*) throughout Europe. Despite numerous reports from Central, Northern, and Southern Europe, its occurrence in South-Eastern Europe has remained largely undocumented. This study provides the first molecularly confirmed report and the first systematic epidemiological assessment of *O. flexuosa* in red deer in Croatia. During the 2024–2025 hunting season, 110 legally harvested red deer from central Croatia were examined for subcutaneous nodules. Nodules were evaluated morphologically, and adult nematodes were identified and confirmed by sequencing of a fragment of the mitochondrial *cytochrome c oxidase subunit I* (*COI*) gene. Subcutaneous nodules were detected in 53.6% (59/110) of examined animals. *O. flexuosa* was confirmed in 52 deer, corresponding to an overall prevalence of 47.3%. Co-infection with *Hypoderma diana* occurred in 21.2% of infected animals. Sequence similarity ranged from 96.37% to 99.85% compared to published European *O. flexuosa* isolates. Phylogenetic analysis placed Croatian sequences within the established European lineage, without evidence of regional genetic divergence. The observed prevalence falls within the intermediate range reported across Europe and indicates stable local transmission. These findings close an important geographical knowledge gap and demonstrate that nodular onchocercosis is established in red deer populations in South-Eastern Europe.

## 1. Introduction

The Onchocercidae comprise 88 genera and represent an important group of vector-borne spirurid nematodes with worldwide distribution [1,2]. The genus *Onchocerca* includes more than 30 species of filarial nematodes transmitted by hematophagous dipteran vectors [3]. Most species parasitize wild and domestic ungulates, in which adult worms inhabit subcutaneous connective tissues, while microfilariae are distributed within the skin, enabling transmission to blood-feeding vectors.

In European red deer (*Cervus elaphus*), four species of the genus *Onchocerca* have been described: *O. flexuosa* Wedl, 1856, *O. jakutensis* Gubanov, 1964, *O. skrjabini* Rukhlyadev, 1964, and *O. garmsi* Bain & Schulz-Key, 1976 [4]. These species differ in anatomical localization and pathological presentation. Adults of *O. flexuosa* share overlapping predilection sites with *O. jakutensis*, forming subcutaneous nodules primarily in the dorsal region, extending from the interscapular area to the lumbar region. In contrast, *O. skrjabini* and *O. garmsi* occur freely within subcutaneous connective tissues and do not form well-defined nodular structures [4]. Infection intensity varies considerably, ranging from only a few nodules to several dozen per individual, depending on ecological conditions, host-related factors, and transmission dynamics.

*Onchocerca flexuosa* is widely distributed across Europe; however, reported prevalence shows pronounced spatial heterogeneity, likely influenced by ecological characteristics, host density, vector abundance, and methodological differences among studies.

Previous reports of *O. flexuosa* in red deer originate predominantly from Central, Northern, and Southern Europe, where the parasite is recognized as an established component of cervid parasitic fauna. Reported prevalence varies substantially between regions and investigations [5], ranging from 5% in the Czech Republic [6] to 96% in Germany [7]. Intermediate prevalence levels have been documented in Romania (13%) [8], Poland (60%) [9], Ukraine (33%) [10], and Belarus (63%) [11]. In Southern Europe, studies from Spain have demonstrated marked regional variation, with prevalence estimates of 24% [12] and 33% [13] in central regions, compared to 85% in northwestern populations [5]. In Northern Europe, a prevalence of 56% has been reported in Sweden [14], while Denmark showed 30.9% [15]. In Central Europe, recent data from Slovakia indicate a prevalence of 47.06% [16].

In contrast, data from South-Eastern Europe remain scarce. To date, no systematic investigations have been conducted in this region. A single historical case was reported from Krim Hill near Ljubljana (former Yugoslavia) in 1967 [17], but without molecular confirmation. Consequently, the presence, prevalence, and epidemiological relevance of *O. flexuosa* in South-Eastern European red deer populations have remained largely undocumented.

Additionally, subcutaneous nodules caused by *O. flexuosa* may be morphologically confused with lesions induced by *H. diana*, potentially leading to historical misclassification. The lack of molecularly confirmed data from this part of Europe therefore represents a significant gap in our understanding of the species’ continental distribution. Accordingly, the present study aimed to determine the prevalence of *Onchocerca* spp. in red deer from Croatia using combined morphological and molecular approaches, and to interpret the findings within the broader European epidemiological context.

## 2. Materials and Methods

### 2.1. Study Region

**Figure 1:**
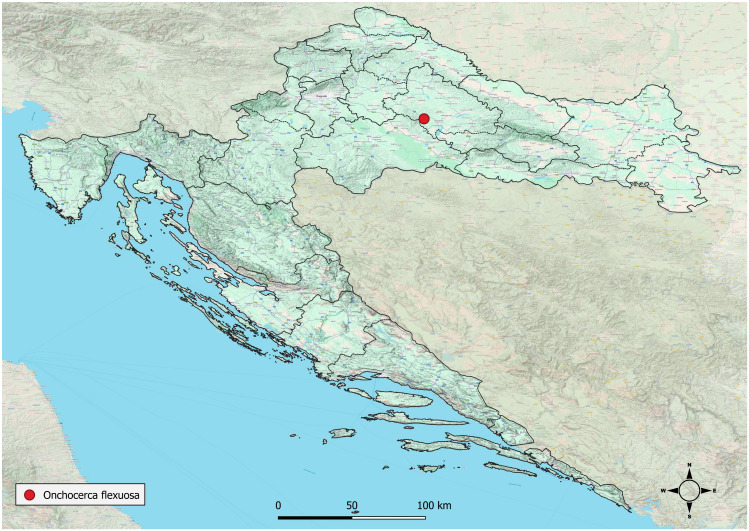
Geographic location of the study area in central Croatia. The red dot indicates the open state hunting ground “Zapadna Garjevica” on the Moslavačka gora massif, where red deer were sampled.

The study was conducted in the open state hunting ground “Zapadna Garjevica”, located on the central massif of Moslavačka gora in central Croatia (Figure 1). Altitude ranges from 120 to 487 m a.s.l., with approximately half of the area situated below 200 m. The terrain is predominantly gently undulating to hilly, intersected by shallow to moderately deep gullies, with occasional steeper slopes reaching inclinations of up to 45°.

Land cover is dominated by forests (~19.650 ha), primarily state-owned and interspersed with agricultural mosaics along peripheral zones. Forest vegetation consists mainly of mixed deciduous stands, including beech (~40%), sessile oak (~20%), hornbeam (~20%), and pedunculate oak (~10%), with smaller patches of conifers and black locust.

The hydrographic network includes numerous permanent small streams and watercourses draining into the Česma River basin, as well as a small reservoir in Podgarić (~4 ha). According to the Köppen classification, the climate is temperate oceanic (Cfb) with continental characteristics. Long-term meteorological data from the nearby Bjelovar station indicate a mean annual temperature of approximately 10.4 °C, with monthly averages ranging from 0.1 °C (January) to 20.4 °C (July), and an average annual precipitation of approximately 839 mm, with a summer maximum. These environmental conditions, together with extensive forest cover and permanent water sources, create favorable habitats for hematophagous dipteran vectors. The hunting ground sustains stable populations of large ungulates. The red deer (*Cervus elaphus*) population was estimated at 840 individuals, corresponding to an approximate density of 3.26 individuals per km^2^ (0.0326 individuals/ha). Roe deer (*Capreolus capreolus*) and wild boar (*Sus scrofa*) are also present in lower numbers.

Between 1 December 2024 and 28 February 2025, a total of 110 legally hunted red deer were included in the study during the regular hunting season, in accordance with national wildlife management regulations. Immediately after skin removal, each carcass was systematically examined for the presence of subcutaneous nodules. For each animal, the number, approximate size, and anatomical localization of nodular lesions were recorded. Nodules were excised, fixed in 96% ethanol, and transported under refrigerated conditions to the laboratory for further parasitological examination.

### 2.2. Morphology

In the laboratory, subcutaneous nodules were dissected under a Stereo Discovery 20 microscope (Zeiss, Jena, Germany). Fibrous capsules were carefully incised, and adult nematodes were extracted using fine forceps and rinsed in sterile physiological saline solution. Morphological identification was based on established anatomical criteria [4]. Particular attention was paid to differentiating *O. flexuosa* from lesions caused by *Hypoderma diana*, which may present with superficially similar subcutaneous nodules. Detailed morphological examination was performed using an Axio Imager M.2 microscope (Zeiss, Jena, Germany) equipped with Axiovision and ZEN 2 Pro software (blue edition, version 3.5.093.00010, Zeiss).

### 2.3. Molecular Analysis

Twenty adult nematodes were preserved in 96% ethanol and stored at −20 °C until molecular analysis. DNA was extracted individually from adult worms using the DNeasy Blood & Tissue Kit (Qiagen, Hilden, Germany) according to the manufacturer’s instructions. Extraction controls were included to monitor potential contamination.

Molecular identification was performed by amplification of a fragment of the mitochondrial *cytochrome c oxidase subunit I* (*COI*) gene. The *COI* fragment was amplified using primers COIintF and COIintR [18] in 20 μL PCR reactions containing 1 μL of DNA template, 0.4 μL of each primer (10 pmol/μL), 10 μL of GoTaq^®^ G2 Master Mix (Promega, Madison, WI, USA), and 8.2 μL of nuclease-free water. Positive and negative controls were included in each PCR run.

Amplified products were analyzed using the QIAxcel capillary electrophoresis system (Qiagen, Hilden, Germany).

PCR products of the expected size were purified using the ExoSAP-IT^®^ kit (Thermo Fisher Scientific, Waltham, MA, USA) and sequenced in both directions using the same primer pair by Macrogen (Amsterdam, The Netherlands). Raw sequences were assembled and edited using SeqMan Pro 18 and SeqBuilder Pro 18 within the Lasergene 18.6 software suite (DNASTAR, Madison, WI, USA).

### 2.4. Phylogenetic Analysis

All publicly available *COI* nucleotide sequences of *Onchocerca* species reported from cervid hosts were retrieved from the GenBank database. Only sequences with unambiguous species identification and sufficient length were retained for further analysis. Multiple sequence alignment was performed using the ClustalW algorithm [19], implemented in BioEdit v7.2.5 [20] under default settings. The resulting alignment was carefully inspected and manually trimmed to ensure uniform sequence length (578 bp).

The most appropriate nucleotide substitution model was selected based on the corrected Akaike Information Criterion (AICc). The Tamura–Nei model with a gamma distribution of rate heterogeneity (TN93 + G) was identified as the best-fitting model (AICc = 4144.545) and was subsequently applied for phylogenetic reconstruction. Phylogenetic relationships were inferred using the Maximum Likelihood (ML) method implemented in MEGA v11 [21]. Branch support was assessed by nonparametric bootstrap analysis with 1000 replicates.

## 3. Results

**Figure 2:**
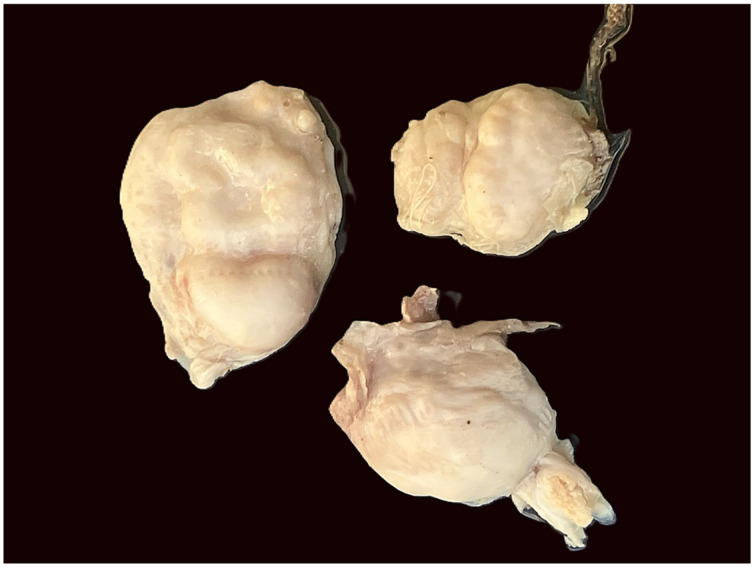
External macroscopic appearance of subcutaneous nodules attributed to *Onchocerca flexuosa*. The nodules are well-demarcated, firm, oval to irregularly rounded structures with a smooth to slightly lobulated surface and pale whitish coloration. A distinct fibrous contour separates the nodules from the surrounding subcutaneous tissue.

Subcutaneous nodules were detected in 59 of 110 examined red deer (53.6%) (Figure 2). The number of nodules per affected animal ranged from one to eleven, with most individuals presenting between two and five nodules. Nodules were consistently located in the dorsal and lumbar regions.

**Figure 3:**
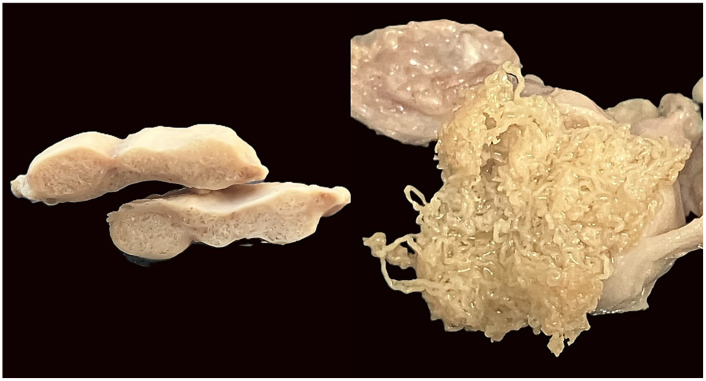
Macroscopic cross-section of a subcutaneous nodule caused by *O. flexuosa*. After incision, a thick fibrous capsule enclosing a compact central mass is visible. The internal structure appears dense and multilayered, corresponding to tightly aggregated adult filarial nematodes embedded within connective tissue.

**Figure 4:**
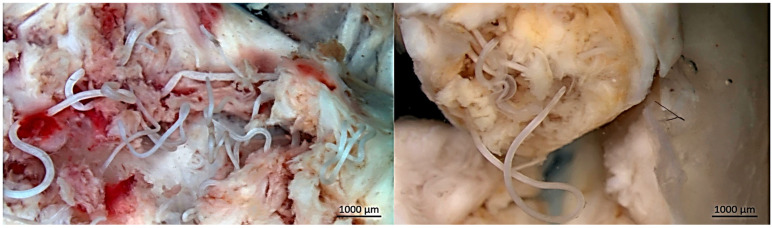
Detailed view of the nodular cavity following incision, showing multiple elongated, slender, whitish adult nematodes of *O. flexuosa*. The worms are tightly coiled and partially embedded within fibrous and inflammatory host tissue. Focal hemorrhagic areas surrounding the parasites reflect local tissue reaction.

**Figure 5:**
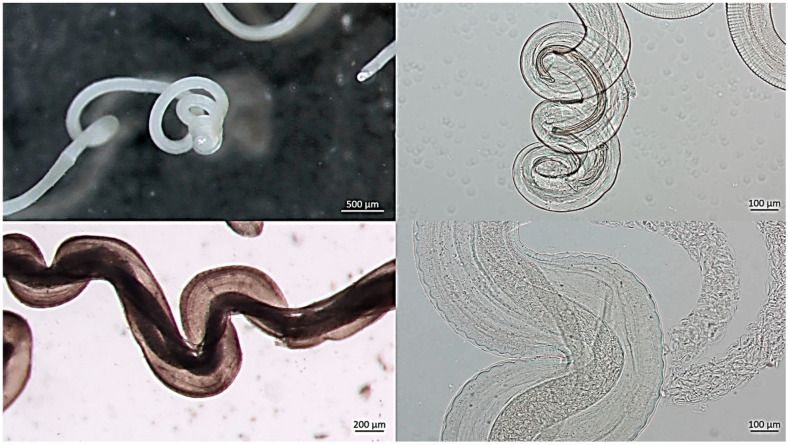
Upper panel: posterior extremity of a male worm showing a gradually tapering body ending in three tightly coiled terminal spirals, representing a distinctive morphological feature of the species. Lower panel: posterior region of a female worm, markedly thicker and less tightly coiled, with visible intrauterine microfilariae occupying the body cavity.

Upon dissection, the nodules consisted of fibrous capsules containing coiled filarial nematodes embedded within connective tissue (Figure 3 and Figure 4). Adult worms were slender, whitish, and tightly intertwined within the nodular cavity. Based on morphological characteristics, *O. flexuosa* was identified in 41 of the 59 nodular animals (69.5%) (Figure 5). In seven cases (11.9%), nodules contained larvae morphologically consistent with *H. diana*. Co-infection with both parasites was observed in 11 animals (18.6%), in which distinct nodules attributable to each parasite were present within the same host.

A total of twenty adult nematodes, each originating from a different host, were subjected to molecular analysis. All specimens used for morphological characterization were genetically confirmed as *O. flexuosa*. Sequence analysis of the *COI* fragment demonstrated 96.37–99.85% similarity to published *O. flexuosa* sequences available in GenBank.

**Figure 6:**
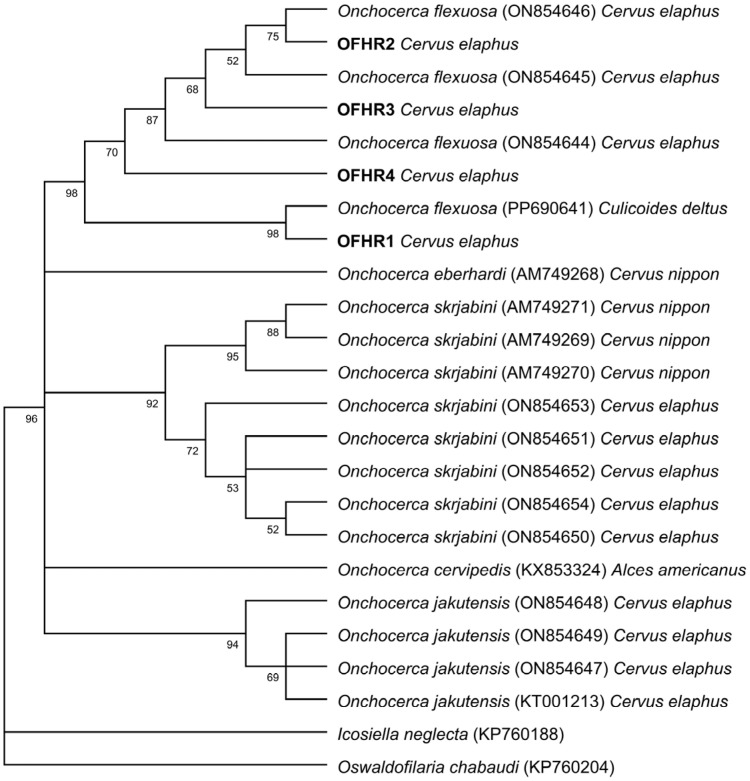
Maximum likelihood (ML) phylogenetic tree showing the relationships among *COI* nucleotide sequences of different *Onchocerca* species infecting cervids. Bootstrap values based on 1000 replicates are shown at the nodes (only values above 50% are displayed). Sequences generated in this study are shown in bold.

Phylogenetic reconstruction revealed that all sequences obtained in the present study clustered within the *O. flexuosa* clade, forming a strongly supported monophyletic group together with reference sequences derived from *Cervus elaphus* (bootstrap = 98%). Within this lineage, Croatian isolates were distributed across several subclusters, indicating detectable intraspecific haplotype diversity. However, no distinct geographic structuring was observed, as Croatian sequences were interspersed with isolates from other European regions. Overall, the phylogenetic analysis confirms the morphological identification of the Croatian isolates as *O. flexuosa* and demonstrates their placement within the broader European cervid-associated lineage (Figure 6).

No amplification was observed in negative extraction or PCR controls. The obtained sequences showed high query coverage and consistent alignment across the analyzed regions. Sequences were submitted to GenBank under the accession numbers PZ050805, PZ050809, PZ050863, and PZ050864.

## 4. Discussion

The present study provides the first systematic and molecularly confirmed investigation of *O. flexuosa* in red deer in Croatia and documents a relatively high prevalence (47.3%) in the examined population. The detection of subcutaneous nodules in more than half of the examined animals (53.6%), together with molecular confirmation of *O. flexuosa* in the majority of nodular cases, indicates that the parasite is firmly established in the investigated area. Its occurrence alongside *Hypoderma diana*, as previously documented [22], further highlights the complexity of subcutaneous parasitic infections in this cervid population.

The prevalence recorded in Croatia falls within the broad European range and closely aligns with prevalence reported from Slovakia (47.06%) [16], Sweden (56%) [14] and Poland (60%) [9]. It differs markedly from both the low prevalence documented in the Czech Republic (5%) [6] and the exceptionally high values reported in Germany (96%) [7] and northwestern Spain (85%) [5]. These comparisons place Croatia within the intermediate European epidemiological corridor, suggesting that South-Eastern Europe shares transmission characteristics with Central European endemic zones rather than representing a marginal area of occurrence.

The substantial variability in prevalence across Europe has been linked to differences in host density, ecological structure, vector abundance, climatic conditions, and methodological design. The ecological features of the Garjevica hunting area—extensive mixed deciduous forests, moderate red deer density (3.26 individuals/km^2^), permanent watercourses, and a temperate continental climate—create favorable conditions for hematophagous dipteran vectors, particularly *Simuliidae* and *Ceratopogonidae* [1]. Such environmental stability likely facilitates sustained parasite circulation.

Although transmission of *O. flexuosa* has traditionally been attributed to biting midges and black flies, direct confirmation of vector species under natural European conditions has been scarce. The recent molecular detection of *O. flexuosa* DNA in *Culicoides deltus* in Slovakia [23] represents a significant advance in clarifying vector involvement. Considering the ecological parallels between Slovak forest ecosystems and central Croatia, it is plausible that similar *Culicoides* species contribute to transmission in the study area. While entomological sampling was beyond the scope of the present investigation, the prevalence observed here is consistent with the presence of competent and locally established vector populations.

Temperature-dependent larval development in dipteran vectors has been demonstrated for other *Onchocerca* species [24,25]. The climatic conditions of central Croatia, including warm summers and sufficient precipitation, likely permit seasonal maturation of infective larvae. When combined with the observed haplotype diversity and absence of phylogeographic segregation, these findings strongly suggest endemic transmission rather than recent introduction or expansion.

Co-infection with *H. diana* in 21.2% of *O. flexuosa*-positive animals represents an important diagnostic consideration. The morphological resemblance between nodules caused by *O. flexuosa* and those induced by *Hypoderma* spp. highlights the potential for underdiagnosis of nodular onchocercosis in wildlife monitoring programs. The integration of morphological examination with molecular confirmation, as applied in this study, therefore provides a more accurate estimate of parasite prevalence and may help reassess historical data.

Phylogenetically, Croatian isolates clustered within the broader European cervid-associated *O. flexuosa* lineage without forming a distinct regional clade. The lack of geographic structuring indicates genetic connectivity across European red deer populations and supports the concept of a transregional transmission network maintained by mobile wildlife hosts and shared ecological conditions. The presence of multiple haplotypes within a single hunting area further supports long-term parasite persistence.

This study is limited by its focus on a single hunting ground and a defined seasonal period, which precludes broader spatial and temporal inference. In addition, the absence of entomological data prevents direct confirmation of local vector species. Future studies integrating expanded geographic sampling, longitudinal monitoring, and vector surveillance will be essential to clarify transmission dynamics and potential climatic influences in South-Eastern Europe.

## 5. Conclusions

In conclusion, this study provides the first molecular confirmation of *O. flexuosa* in Croatian red deer and demonstrates that the parasite is firmly established in South-Eastern Europe. The observed prevalence and phylogenetic placement align Croatia with recognized Central European endemic zones, thereby extending the confirmed distribution range of the species. These findings underscore the importance of integrating morphological and molecular approaches in wildlife parasitology and highlight the need for continued ecological and vector-based investigations to fully elucidate the epidemiology of nodular onchocercosis in European cervids.

## Data Availability

The original contributions presented in this study are included in the article. Further inquiries can be directed to the corresponding author.
